# Effect of screw angulation and multiple insertions on load-to-failure of polyaxial locking system

**DOI:** 10.1371/journal.pone.0295526

**Published:** 2023-12-11

**Authors:** Jakub Glowacki, Tomasz Bartkowiak, Piotr Paczos, Patryk Mietlinski, Pawel Zawadzki, Lukasz Lapaj

**Affiliations:** 1 Department of General Orthopaedics, Musculoskeletal Oncology and Trauma Surgery, Poznan University of Medical Sciences, Poznań, Poland; 2 Institute of Mechanical Technology, Poznan University of Technology, Poznań, Poland; 3 Institute of Applied Mechanics, Poznan University of Technology, Poznań, Poland; Gdańsk University of Technology: Politechnika Gdanska, POLAND

## Abstract

**Purpose:**

Polyaxial locking plates rely on the alignment between the thread-to-thread connections of the screw head and the plate hole. These implants have provided substantial support for surgeons. In particular, extended screw positioning have proven to be beneficial in the fixation of challenging fractures. This study aimed to investigate the mechanical properties of ChM 5.0 ChLP polyaxial screws inserted in off-axis trajectories, including multiple insertions and to correlate these parameters with the screw head and the plate hole thread-to-thread engagement.

**Methods:**

Polyaxial locking screws were inserted into the plates at various angles (0°,10°,15°, -15° off-axis). Multiple time inserted screws were placed firstly at 15°, then 0° and finally -15° off-axis in the same plate hole. A microCT scan of the plate-hole and screw-head interface was conducted before destructive tests. Representative screws from each group were also examined by Scanning Electron Microscope.

**Results:**

The standard insertion at 0° sustained the greatest maximum bending strength without relocation in the screw hole. Screws inserted at 10° and 15° (one time) showed a significant reduction in load-to-failure of up to 36% and 55%, (p = 0.001) (p = 0.001) respectively. Screws inserted at -15° after a maximum of three multiple insertions with angle shift, showed a total reduction in force of up to 70% (p = 0.001). A microCT analysis of thread engagement showed significant correlations. However, the results obtained for multiple insertions were highly variable.

**Conclusions:**

ChM 5.0 ChLP polyaxial locking system has valuable properties that foster fracture fixation, providing various surgical options. Nevertheless, the freedom of off-axis placement and multiple insertions of the screws comes at the price of reduced force. When possible surgeons should minimize the angles of insertions.

## Introduction

Locking plates were introduced to clinical practice in 2000 and are currently the gold standard for fixation of various types of fractures [1]. They rely on the engagement of threaded screw heads into the threaded plate holes, thus creating a reliable construct that provides a robust fixation of bone fragments [2]. In conventional designs, precise alignment of both the screw and the plate is critical for the stability of the construct. Even minor angle deviations substantially decrease the load to failure. Some studies suggest that a variation of monoaxial screws by 10° could reduce failure load up to 68% [2]. This technical problem was solved by polyaxial locking plates, which allow stable positioning of the screws in plate holes at varying angles [3]. Extended screw positioning angles could address numerous clinical situations in which fracture management involves previously implanted orthopedic devices, such as joint replacement components. The surgeon may need to place the screws in an orientation that differs from the position pre-determined by the alignment of the holes [4]. Various polyaxial locking mechanisms with different loads to failure exist on the market today [3, 5, 6]. The most recognizable mechanisms are: cut-in, locking-cap, expansion bushing, point loading thread-in [5]. Furthermore, some implant manufacturers allow multiple insertions of the screw combined with angle shifting while using the same screw-hole combination. An example of such an approach is the ChM 5.0 ChLP system, which incorporates hard CoCrMO alloy screws engaging into softer titanium alloy plates [7]. In short, it is claimed that a screw can be placed in a given plate hole and removed several times without significantly compromising its stability. This solution might be helpful for surgeons, as it could address various clinical situations, such as insufficient bone stock or screw malposition [5].

However, using multiple insertions still raises concerns in the orthopedic community, as there is little data on the stability of such a construct. To our knowledge, the mechanical assessment of multiple passes of the screws combined with an angle shift has not been reported yet. Most of the mechanical studies investigating locking plates focused on the torsional and axial compression of the plates or the bone-plate constructs [6, 8–10]. Few studies analyzed and compared the connection strength between the screw head and the plate hole [11, 12]. This seems to be a weak point in polyaxial designs, as the engagement between components is limited and multiple insertions can further compromise it. Surprisingly, most studies only investigated the interface between the screw head and the plate hole using destructive tests [11–14]. Consequently, little is known about the engagement of the screw head and the plate hole components in cases of single and multiple insertions of the screws. In the ChM 5.0 ChLP system, where the thread of a hard screw head can cut into the softer material of plate holes, it can be assumed that placing polyaxial screws off-axis would likely result in the reduced contact area between the screw head and plate hole. This area could be reduced further by multiple insertions of a screw at varying angles. As the interface between components is responsible for the strength of the mechanical connection, the amount of thread engagement would significantly correlate with the mechanical parameters of the screw head and the plate hole interface.

This study aimed to analyze the amount of thread connection between the screw head and the locking plate hole of the polyaxial plate construct using a metrological X-ray micro-computed tomography (microCT). The second objective of this study was to test the mechanical properties of the ChM 5.0 ChLP polyaxial locking mechanism with screws placed at various angles from 0° to 15°, including multiple insertions.

## Material and methods

### Plates and screw placement

**Fig 1:**
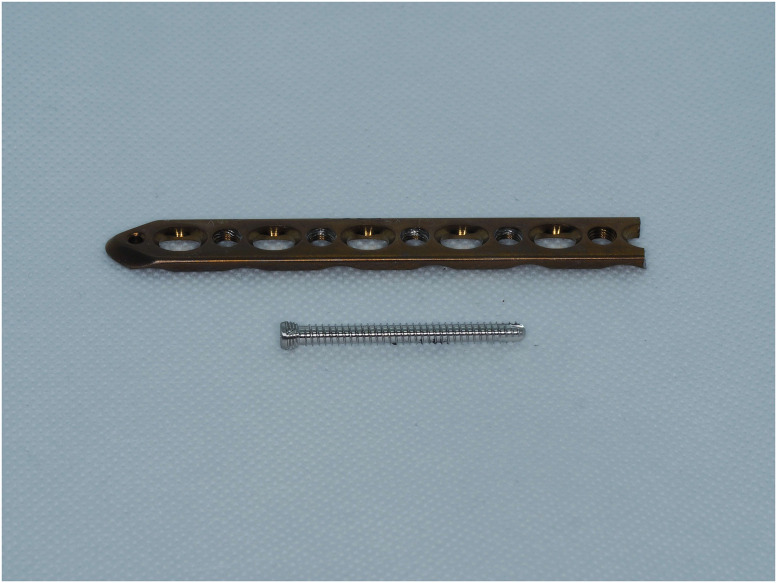
The ChM 5.0 ChLP plate and 3,5 mm variable angle locking screw.

**Fig 2:**
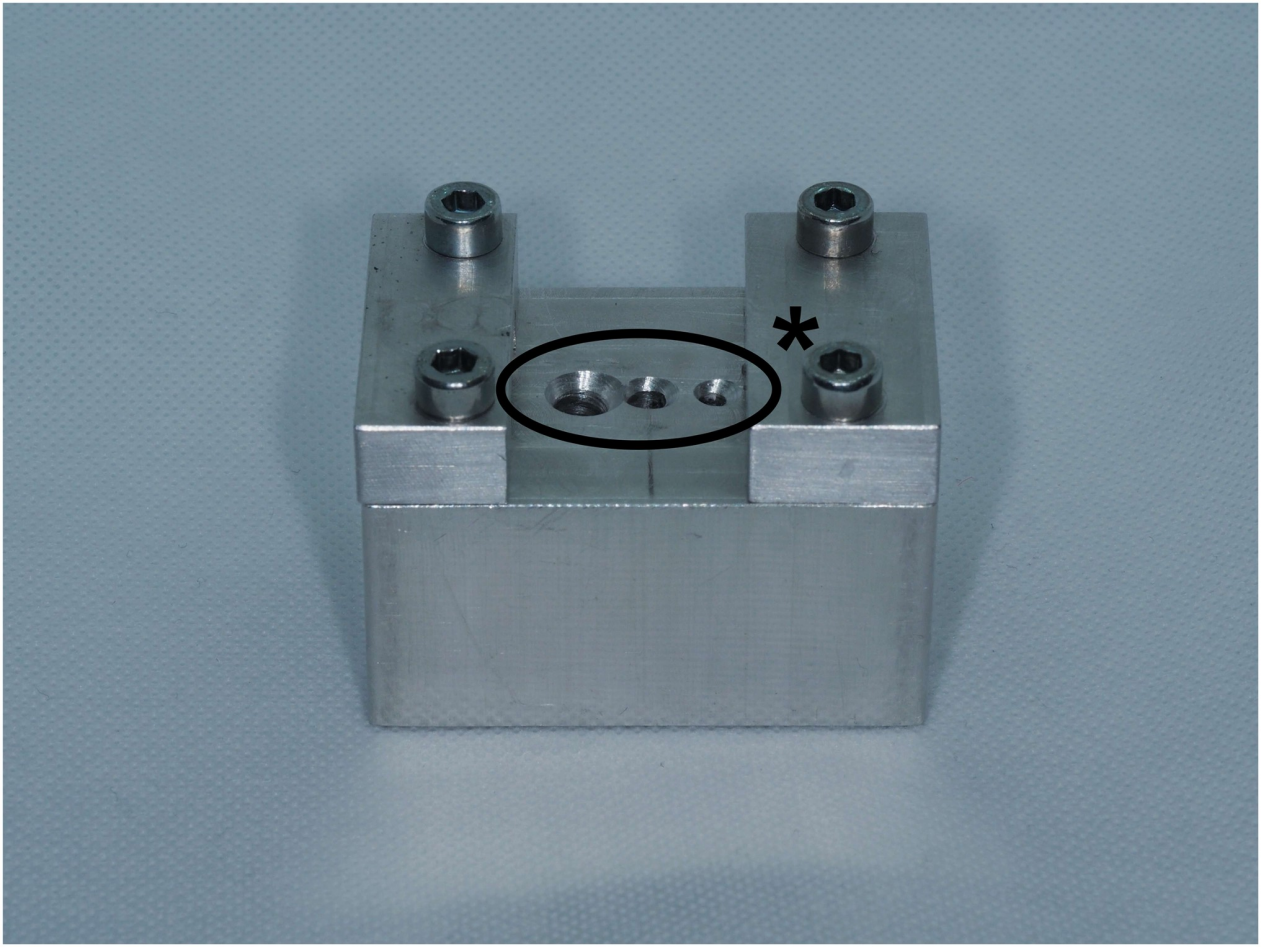
Pre-formed angulated block. * holes prepared for different diameters of the screws.

**Fig 3:**
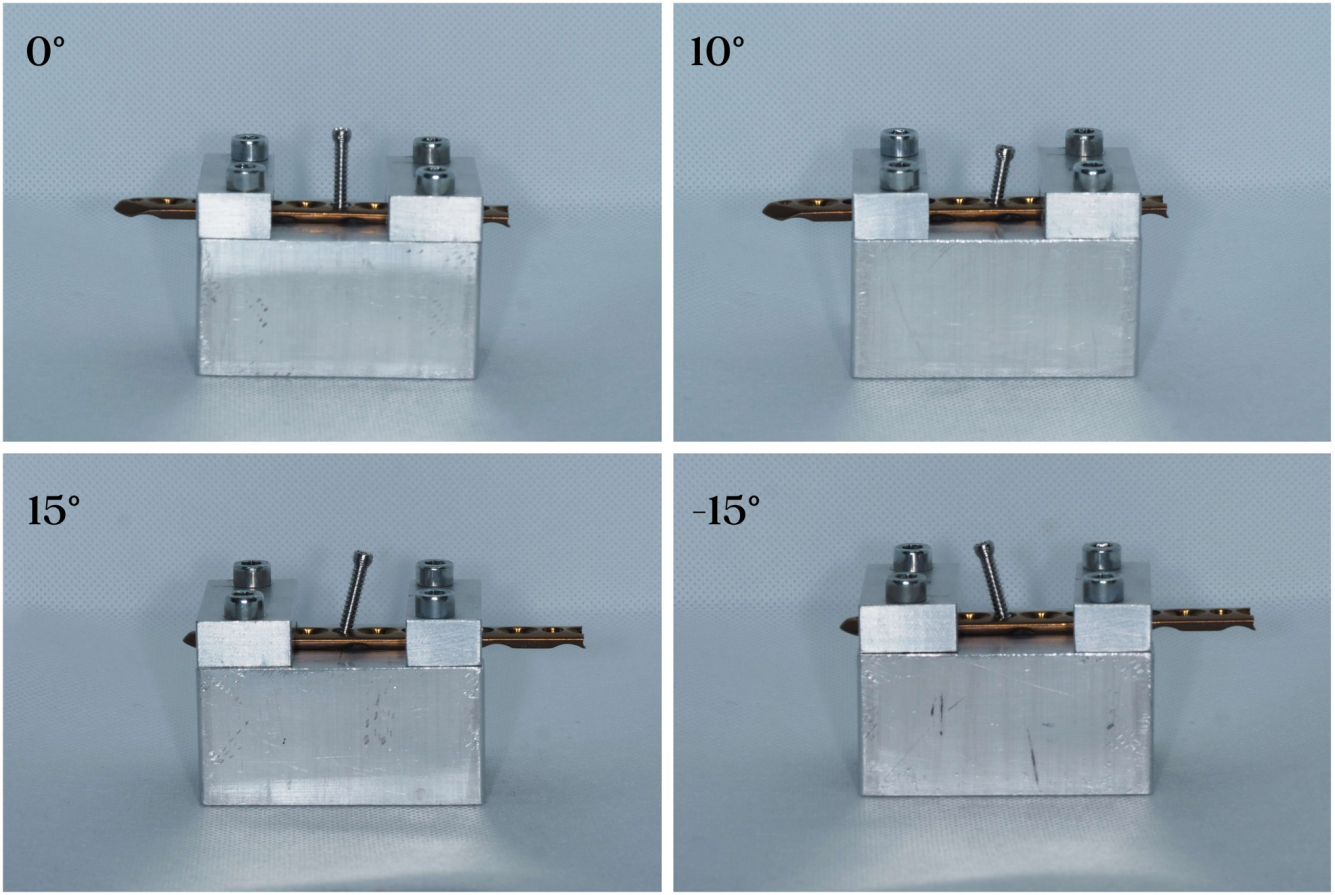
Four types of setups. Analysis of the thread-to-thread contact regions.

This study examined components from the ChM 5.0 ChLP polyaxial locking system (ChM Sp. z o.o., Lewickie, Poland). The system consists of plates made of titanium alloy (ISO 5832-2/ASTM F67) and 3,5 mm variable angle locking screws made from CoCrMo alloy (ISO 5832-12/ASTM F1537) (Fig 1) [7]. The design allows for polyaxial screw insertion at an angle of up to 15° in all directions with the threads of the spherical screw heads engaging into the threaded parts of the cylindrical plate holes. The ChM manufacturer`s instruction also allows the screw to be placed at varying angles up to three times in the same hole [7]. To standardize all samples, first, a pre-formed angulated block facilitating screw placement at angles of 0°, 10° and 15° was machined using a CNC 5-axis milling center (DMU 60 Monoblock, DMG Mori, Bielefeld, Germany) (variance ± 1°) (Fig 2). Subsequently, identical screws were placed into holes with a calibrated torque-limiting screwdriver (MicroClick MC 5, Proxxon Industrial). The resolution of this device is determined by the Scale ring with 0.1 Nm graduation. The manufacturer certified that the accuracy was +/- 6%. The insertion torque applied to each screw on each plate was standardized to 2,0 Nm values recommended by the manufacturer [7]. Four types of setups were prepared: samples with screws placed in a single insertion at 0°,10° and 15°, and samples where the screws were placed in multiple insertions at the same plate-hole–first at 15°, then 0° and finally at -15° in the opposite direction. The final configuration reflects the most unfavorable condition for the screw plate interface at the maximal acceptable range of 30°. Once the limiting torque had been set, it was not readjusted and all the screws were tightened at the same condition by the lead author-certified orthopedic surgeon (Fig 3).

MicroCT was used to quantify the contact area between threaded parts of both the screw head and plate hole, as described by Kaczmarek et al. [15, 16]. To put it concisely, after each construct was created, it was scanned with a metrological X-ray micro-computed tomographic device (v|tome|x s240; GE Sensing & Inspection Technologies GmbH, Wunstorf, Germany). MicroCT scanning was performed at the X-ray beam power of 30.7 W (205 kV/150 μA). The exposition time for one image was 500 milliseconds and the voxel size was 25.169 μm. The measurement resulted in a 3D model of the threaded connection. The model was then converted into a series of 2D images representing cross-sections cut orthogonally to the locking screw axis every 20 μm. In those images, thread engagement could be noticed as a region without a distinctive gap between the screw and the plate. In the proposed method, the engagement or gap was detected through automated image postprocessing. For each image a full connection was assumed where there was no gap between the screw and the plate. Each image was given a score between 0% and 100% depending on amount of engagement calculated through automatic pixel-by-pixel examination of the screw-plate interface region. Average thread engagement (ATE) measure was introduced, which corresponds to the arithmetic mean of the individual scores and it was calculated for all cross-sections. The thread-to-thread contact regions of each screw head were visualized (every 20 μm) in a form of a 3D polar plot.

### Scanning electron microscopy examination

After microCT examinations, selected screws from each sample group were removed, and the threaded connections were examined for damage related to screw placement at different angles. Samples were first sonicated in isopropanol and analyzed using a Philips XL-40 Scanning Electron Microscope (SEM). Images were taken at 20 kV acceleration; imaging obtained using the secondary electron detector (SED) was used to examine the structure of the threaded holes. Backscattered electron detector (BSD) imaging was used to examine the screw because the Z-contrast provided by this technique allows for easy identification of materials on the image. Briefly, in BSD images, structures containing heavier elements (Co, Cr, Mo in this study) were brighter than structures formed from the titanium alloy containing lighter elements.

### Mechanical setup

The bending strength of the polyaxial plates constructs was assessed using a universal servohydraulic testing machine (ZWICK Z100/ TL3S Zwick GmbH & Co. KG, Ulm, Germany), with bending strength measurements reported in Nm. The resolution and accuracy of distance measurement were 1 μm and 2 μm accordingly. For distance, we used a built-in sensor of the testing machine. The force was measured by 5 kN load cell (Xforce HP, Zwick GmbH & Co. KG, Ulm, Germany). The resolution and accuracy of the force measurements were 0.01 N and 1% of the nominal load (accuracy class 0.5). Calibration of the machine was carried out by an external accredited laboratory in 2023.

**Fig 4:**
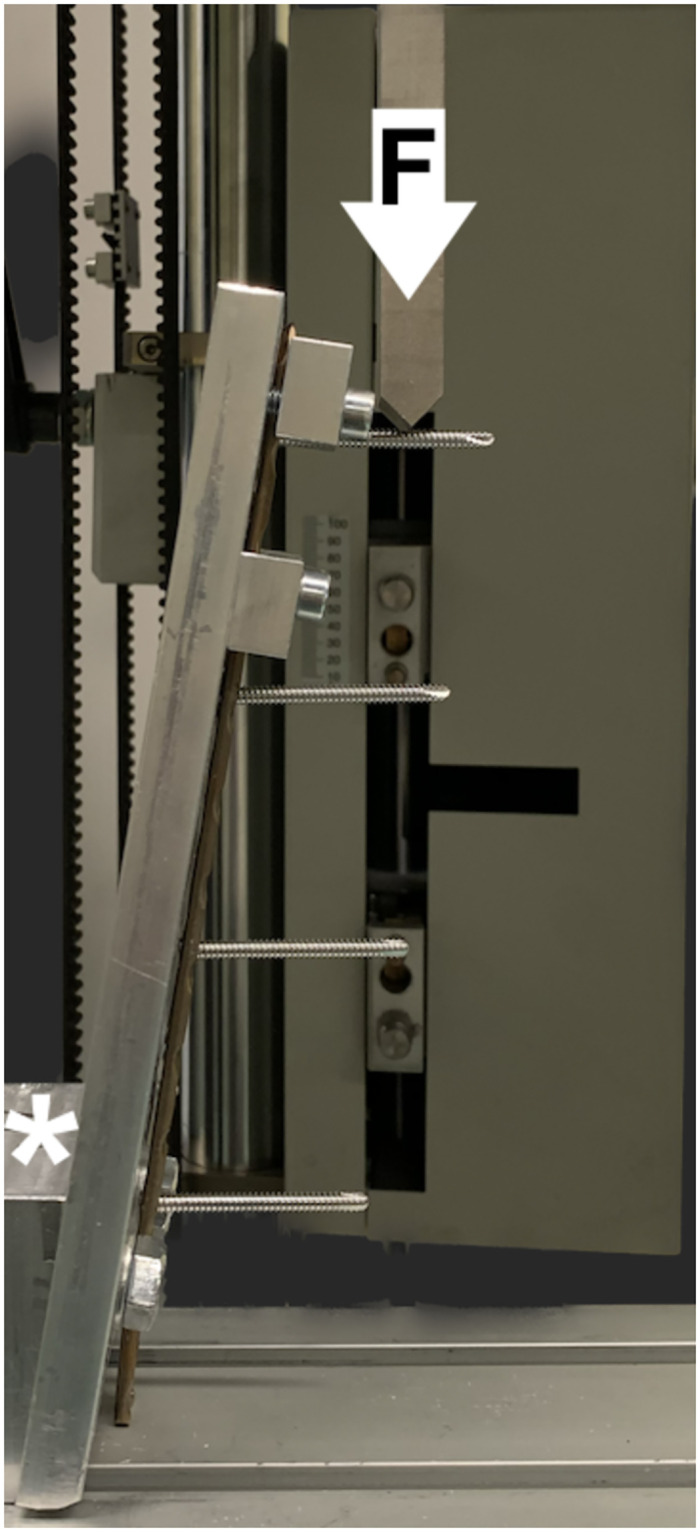
Set-up configuration. *displacement speed 1mm/min.

The plate was clamped in a custom jig with the additional angulated blocks, machined using a CNC 5-axis milling center (DMU 60 Monoblock, DMG Mori, Bielefeld, Germany) (variance ± 1°) (Fig 4). At angles of 0°, 10° and 15° corresponding to the angles of analyzed screws, to establish perpendicular compressive load to the analyzed interface without plate movement. A constant displacement (1 mm/min) was applied at a distance of 21,6 mm from underneath the plate until screw failure occurred (defined as a breakage, rapid loss of >50% force). All measurements adhered to the adapted standards of the American Society for Testing and Materials-ASTM Standard F384-17 [17]. Subsequently, tests were conducted for all inserted screws.

### Statistics

Statistical analyses were performed using Mathematica 12 software (Wolfram Research, Inc., Oxfordshire, United Kingdom). Data were reported as mean±standard deviation, statistical significance was set to p < 0.05.

To assess the impact of the screw insertion on average thread engagement and bending strength, one-way ANOVA was utilized. The normality of residuals was determined using the Shapiro–Wilk test. To investigate the presence of any significant differences between the groups, the post-hoc Tukey test was conducted. A linear regression analysis was performed to determine the strengths of the correlation (R^2^) between ATE and bending strength.

## Results

MicroCT examination demonstrated that placement of the ChM 3,5 mm screws in off-axis trajectories affected the ATE. The initial ATE was the highest for screws inserted at 0° and the lowest for samples inserted at 15° in cases of single insertion. Despite tight fixation, the ATE was lower than 50% for all analyzed configurations. Statistical examination with the ANOVA test demonstrated that the insertion angle was a statistically significant factor affecting ATE (p = 0.0032) (Table 1).

**Table 1:** The relative contact area between the screw head plate-hole for ChM 5.0 ChLP polyaxial system.

	No. of samples	Average thread engagement
**0**°—**one-time locking**	**6**	**47.25%±8.23%**
**10**°—**one-time locking**	**6**	**31.85%±13.00%**
**15**°—**one-time locking**	**7**	**19.38%±14.42%**
**-15**°—**three-time locking**	**7**	**24.46%±6.37%**

**Fig 5:**
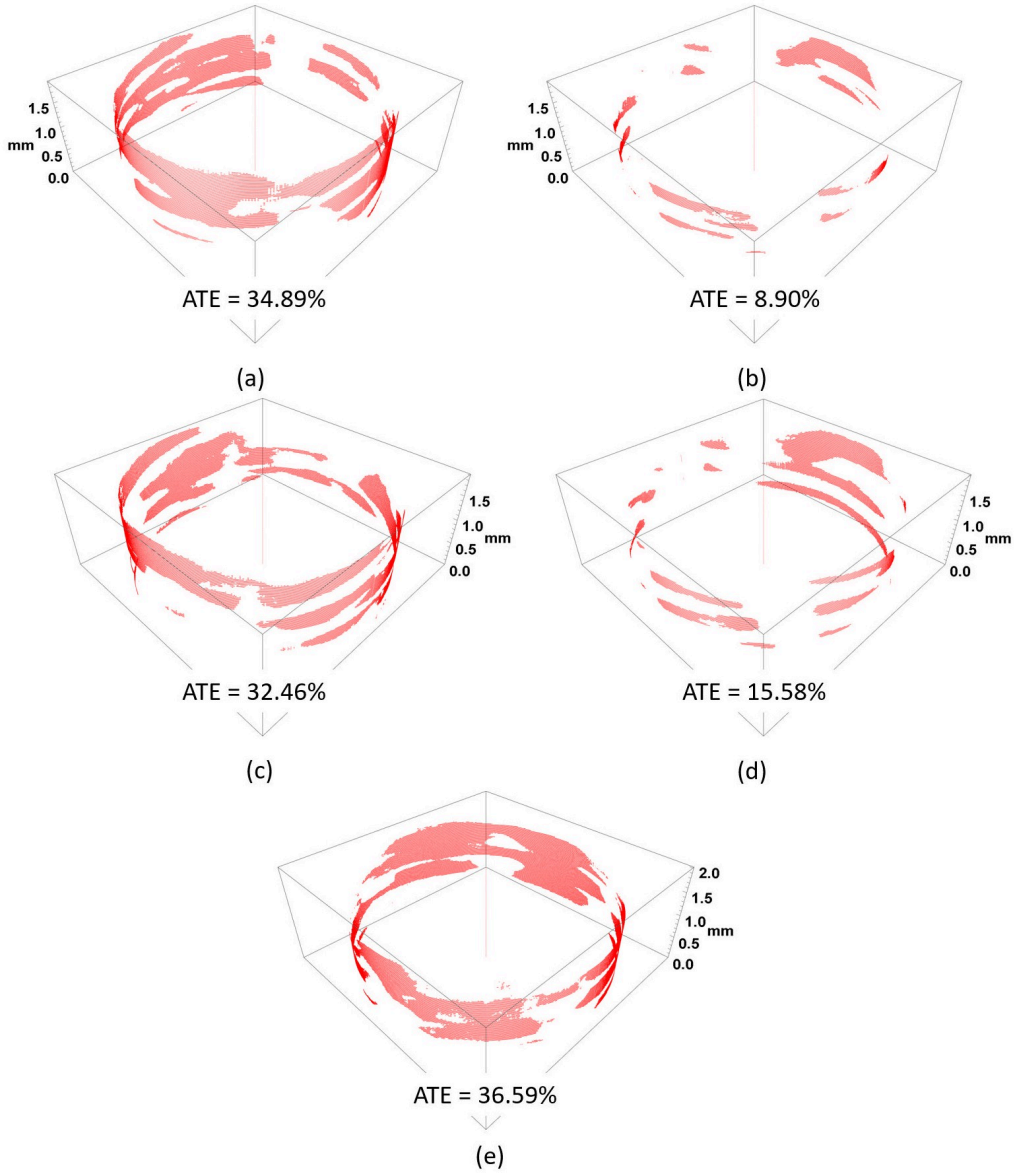
Visualizations of thread engagement in screw-plate interfaces inserted at 15° angle for highest (a) and lowest (b) ATE for single insertion; highest (c) and lowest (d) ATE for multiple insertions. Control group at 0°—single insertion (e).

Surprisingly, the post-hoc Tukey test demonstrated that the screws inserted at 15° after three insertions had sufficient anchoring with no statistically significant differences compared to screws locked at 10° and 15° in a single insertion. Significant differences in the ATE between 0° and 15°, including multiple insertions (Table 2). In general, the results of ATE were highly variable. Fig 5 presents a visualization of the screws placed at 15° with the highest and the lowest ATE.

**Table 2:** P-values (post-hoc Tukey test) for the pair-wise comparison of ATE between insertion angles. Please note that the pairs presented exhibit statistically significant differences.

Pair-wise comparison of the insertion method	p-value	Significance
0° vs 15°	0.001	p<0.01
0° vs -15° -multiple insertions	0.004	p<0.01

**Fig 6:**
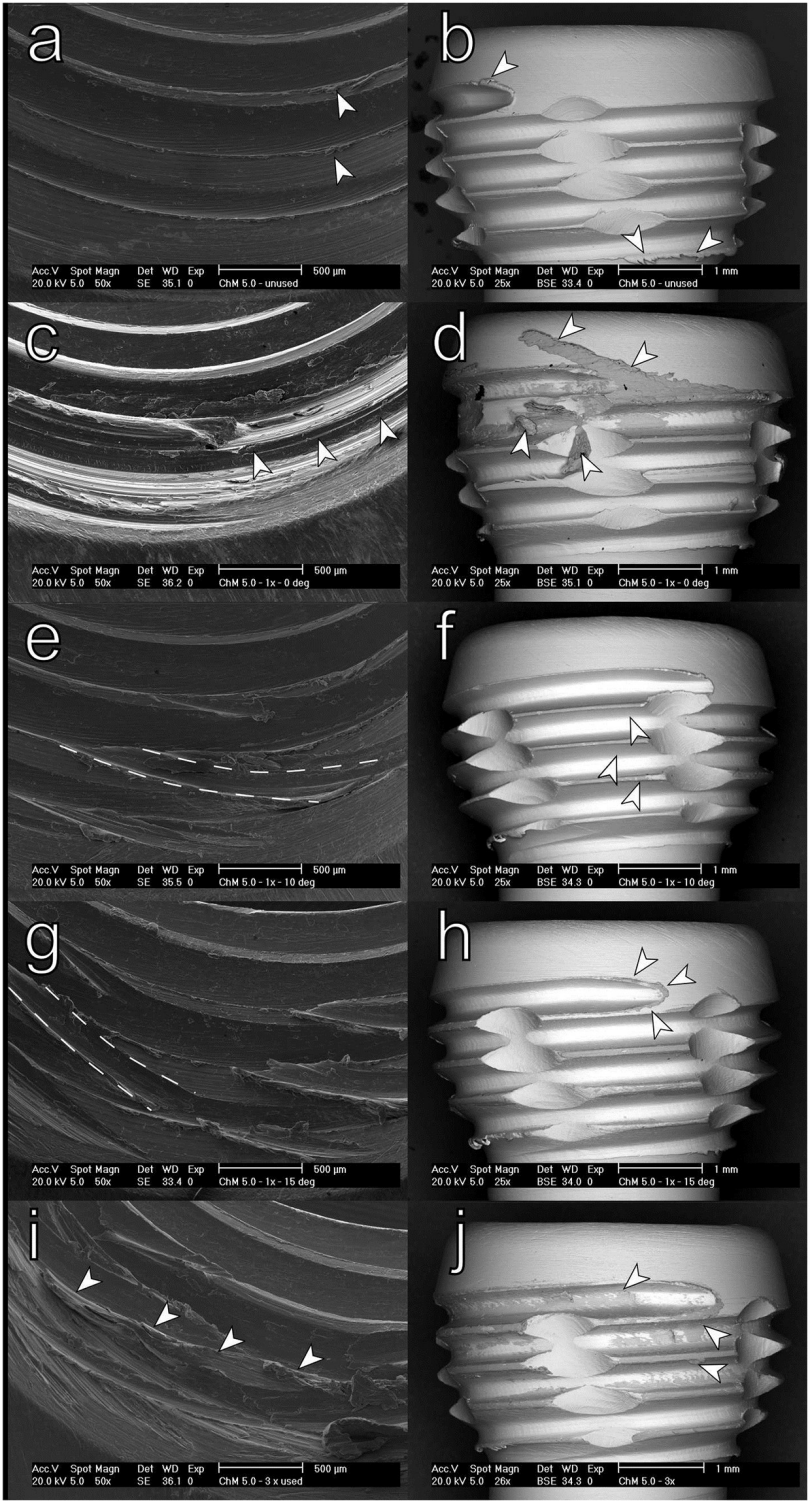
SEM images of disassembled screw-plate constructs. (a) pristine plate hole; arrows indicate small remnants of material after machining (b) unused screw head; arrows indicate small fragments of material after machining (c) plate hole after screw placement at 0°, arrows indicate superficial damage to the proximal part of the thread (d) screw head after insertion at 0°; multiple fragments of plate materials transferred onto the thread as indicated by arrows; (e) plate hole after placement of screw 10° off-axis; cutting of new thread visible; one fragment of new thread indicated by dashed lines; (f) screw head following 10° off-axis placement; arrows indicate small deposits of Ti alloy (g) plate hole after screw insertion at 15°; newly cut thread indicated by dashed line; (h) screw head after insertion at 15°; arrows indicate small patches of transferred plate material; (i) screw hole after three insertions of one screw at 15°,0° and -15°; arrows indicate thread stripping in the proximal part of the hole (j) screw placed in multiple insertions in one hole 15°,0° and -15°; arrows indicate deposits of material stripped from the plate hole.

SEM examination of disassembled components demonstrated similar findings. While the pristine threads of plate holes and screws exhibited only minor machining marks (Fig 6a and 6b), the assembly of the components resulted primarily in plate material damage. In the case of screws placed at 0° SEM, images revealed a direct engagement of both threads. (Fig 6c and 6d), with some thread damage on the outer part of the hole and material transferred onto threads closer to the Torx socket.

In contrast, the placement of screws in a single insertion at 10° and 15° caused the cutting of new threads within the plate hole and limited transfer of material onto the screw heads, which also occurred predominantly on proximal threads (Fig 6e and 6h). In the case of the screws placed in multiple insertions, severe thread damage occurred within stripping of the proximal thread in the plate holes, and noticeable metal transfer onto the screw heads (Fig 6i and 6j).

Initially, a control group comprised of the ChM 5.0 ChLP 3,5mm polyaxial screws inserted at 0° underwent testing. The screws failed without relocation in the screw hole and rapid force loss due to plastic deformation of the screw with macroscopic bending of the screw shaft. A sufficiently strong construct was achievable at 10° and 15° angles—including multiple insertions. In these cases, all screws experienced failure with relocation in the screw hole. A uniform pattern of macroscopic failure occurred in the screw head and plate hole construct specifically in ChM 5.0 ChLP thread-in mechanism, with all screws inserted off-axis. This disfigurement was caused by the plastic deformation of the titanium alloy used for the ChM 5.0 ChLP plate. Mechanical tests demonstrated that the most reliable construct was achieved when screws were placed monoaxially, with its strength decreasing as the insertion angle increased (Table 3). The insertion angle was a statistically significant factor affecting the bending strength (p<0.001).

**Table 3:** Load to failure parameters of the ChM 5.0 ChLP polyaxial system.

Insertion method	No. of samples	Bending strength [Nm]
**0**°**—one-time locking**	**6**	**4.51±0.19**
**10**°**—one-time locking**	**6**	**2.87±0.83**
**15**°**—one-time locking**	**7**	**2.02±0.71**
**-15**°**—three-time locking**	**7**	**1.36±0.38**

**Fig 7:**
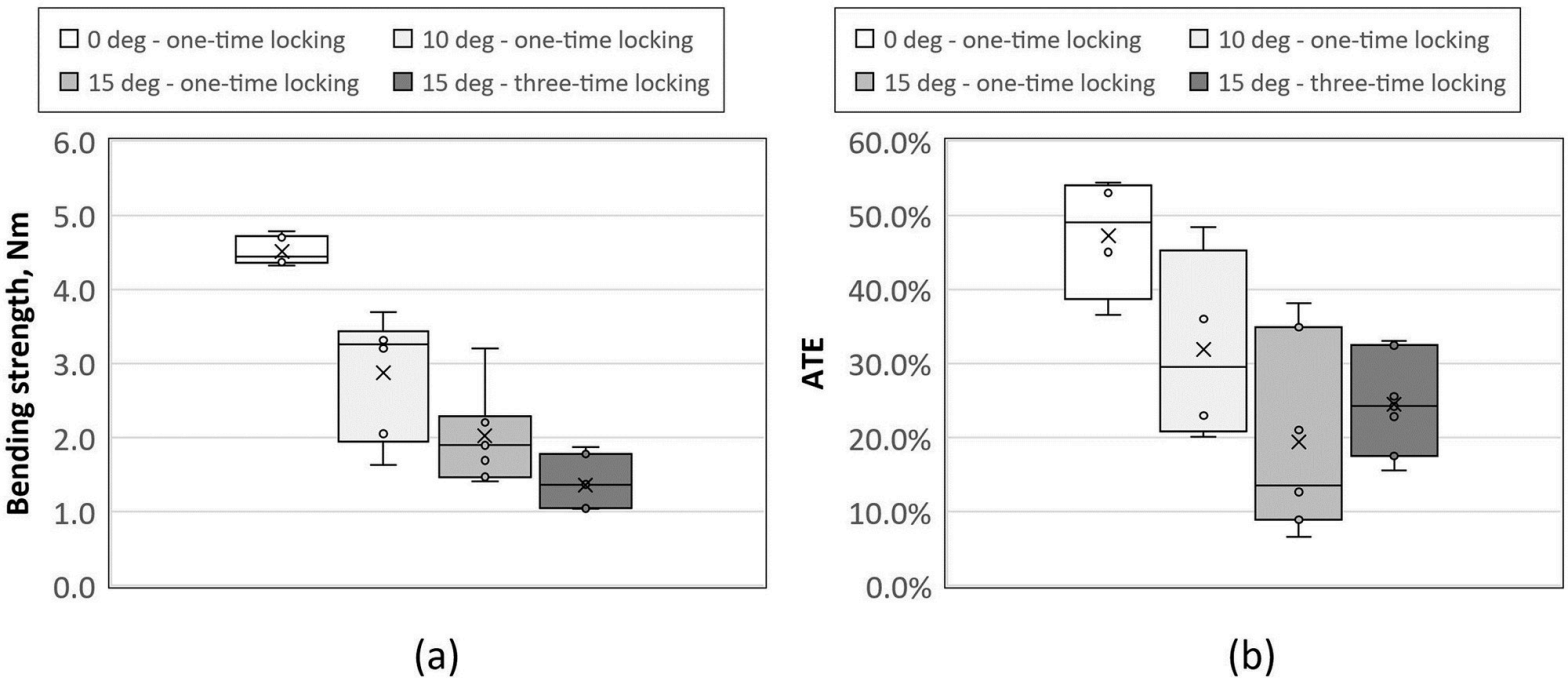
Box and whisker plots of: a) bending strength and b) ATE for all analyzed angles and number of insertions.

Statistical analysis (ANOVA test) demonstrated that the bending strength is affected by the insertion method (p<0.001). Furthermore, the bending strength of screws placed after three insertions was significantly lower than in all single-insertion configurations (Table 4) (Fig 7).

**Table 4:** P-values (post-hoc Tukey test) for the pair-wise comparison of average bending strength between the insertion angles. Please note that the pairs presented exhibit statistically significant differences.

Pair-wise comparison of the insertion method	p-value	Significance
0° vs 10°	0.001	p<0.01
0° vs 15°	0.001	p<0.01
0° vs -15°—multiple insertions	0.001	p<0.01
10° vs -15°—multiple insertions	0.001	p<0.01
15° vs -15°—multiple insertions	0.029	p<0.05

## Discussion

Polyaxial locking screws have many attractive properties compared to monoaxial screws. They allow the surgeon to fix bony fragments in irregular orientations, proving particularly useful in managing periprosthetic fractures where conventionally oriented screws may clash with the existing implant [18]. However, this flexibility bears certain limitations. MicroCT scan data demonstrated that the engagement was most complete in cases where the screw was placed axially, while off-axis placement resulted in reduced thread engagement between the plate hole and the screw head. This data was consistent with SEM observations, which revealed that off-axis insertion resulted in cutting the new thread in the existing thread of the plate hole. When deviations from the axis were slight, a new thread was cut almost perpendicular to the original, allowing for a relatively large contact area between the screw and plate. However, as the deviations increased, less material could be cut, resulting in a decreased ATE.

Within the ChM 5.0 ChLP system, thread engagement occurred predominantly in the proximal part of the holes (near the outer plate surface) and on the proximal part of the head (near the Torx socket). This was attributed to the cylindrical geometry of the holes and the spherical shape of the heads. Consequently, during initial placement, thread contact occurred primarily in the proximal region of both components. However, as the plate hole was damaged through multiple screw insertions, this geometry allowed for cutting new threads in more profound parts of the hole, which can explain why ATE was relatively high in these cases. Noteworthy, the analyzed bending strength deteriorated significantly (p = 0.029) compared to single insertion at 15° off-axis. Therefore, the official manufacturer`s approval for multiple insertions should be utilized exceptionally [7].

Numerous biomechanical studies about polyaxial plates evaluate them in an environment similar to the clinical application, often considering the entirety of the fracture model with multiple inserted screws [6, 8, 18]. Some of these studies proved no statistically significant difference between standard monoaxial fixation and polyaxial fixation [4, 6, 19]. Studies conducted with composite bone constructs have demonstrated similar stability of polyaxial and monoaxial plates [9, 20]. Other studies utilized formalin-fixed or frozen bone specimens with variable bone stock, raising concerns about reproducibility [8, 19]. Studies concerning the analysis of polyaxial fixation in a clinical context give inconsistent results. Tank et al. proved that early mechanical failures with the Synthes variable angle locking plates are more frequent than traditional monoaxial plates [21]. On the other hand, Hanshen et al. indicated improved functional and radiological outcomes in patients treated with Zimmer non-contact bridging polyaxial plate (NCB) [22]. Zhang et al. hypothesized on the basis of the finite element surgical model, that inserting the proximal polyaxial screws at -10° in the transverse plane may reduce Zimmer NCB implant failure [23]. It is worth mentioning that, in particular, in the Zimmer NCB, the locking cap placed over the screw head provides the friction fit, creating fixed angle construct [5]. In turn, the implants discussed in this study demonstrate different styles of screw plate interface, which rely on interdigitation between the threaded screw head and the threaded screw hole.

Few studies verified specifically those threaded connections in the context of the individual screw failure [3, 24]. Yet, the wide range of mechanical tests, varying plate thicknesses, different locking mechanisms and various metal alloys used, direct comparisons of bending strength obtained in the tests between polyaxial systems should be treated with extreme caution. In a study by Hebert-Davies et al., investigation into three small-fragment polyaxial plates with distinct locking mechanisms revealed reductions in maximal force of up to 45% and 34% for Stryker VariAx plates with 3,5mm screws and Smith&Naphew Peri-Loc with 3,5mm screws, respectively [3]. This drop of force was recorded between polyaxial screws inserted monoaxially and 15° off-axis [3]. Similarly to the analyzed 5.0 ChM ChLP and 3,5 mm screws, Stryker VariAx 3,5 mm screws inserted off-axis also failed at the softer plate. Mehling et al. examined three distal radius polyaxial systems under cyclic loading conditions. Bending of the screw shaft was the most common scenario with an insertion angle of 0°. The failure of the locking system occurred with an insertion angle of 10° or more [6]. The results are coherent with our microCT analyses, where ATE for ChM 5.0 ChLP and 3,5mm screws was 47% with an insertion angle of 0°, and was reduced to 19% with off-axis placement 15°. Thus, the strength of the locking mechanism was superior to the screw durability at 0° which resulted in the bending of screws placed at this angle. Mehling et al. reported that the mean bending moment at failure was 1.2 Nm at 0° and 0,8 Nm at 15° in all tested systems [6]. Although these mean values are lower than those presented in this study, it is crucial to acknowledge that these tests were performed under cycling loading with various implant thicknesses throughout the tests [6]. In contrast, during testing of the Stryker VariAX plate and 3,5mm screws, the bending moment at failure actually increased up to the insertion angle of 10°[6]. However, it is not the case in the ChM 5.0 ChLP plate and 3,5mm screws, where the loss of ATE correlated with higher insertional angles. Hoffemeier et al. observed a reduction of ultimate strength of up to 43% and 50% for the screws inserted at 10° and 20°, respectively [25]. In our study, we observed only a 36% drop of bending strength for ChM 5.0 3,5 mm screws inserted at 10°. However, for the screws inserted at 15°, the drop in bending strength was up to 55%.

The incorporation of new polyaxial designs introduces additional costs, warranting a thorough verification of this system’s superiority. Moreover, surgeons tend to prefer mechanisms with similar strength, disregarding the desired angle or multiple insertions through the same plate hole. Current research and our findings proved that this is not the case yet. A comprehensive understanding of the mechanical parameters of each implant is essential for surgeons’ everyday practice.

This study exhibits several limitations. Firstly, the sample size is relatively small. Secondly, multiple insertions of the screws and thus, contamination of screw head-plate hole interface with titanium alloy particles could have affected our microCT analysis, potentially leading to an overestimation of ATE for multiple insertions. Moreover, our analysis focused exclusively on individual screw failures within one type of variable angle fracture fixation system. Finally, many variables could introduce biases to the mechanical parameters, including not macroscopically visible plastic deformation of the plate-screw construct or setup construct.

## Conclusions

Regarding the effect of the screw locking angles, the presented study provides consistent results with practical clinical implications. It was demonstrated that ChM 3,5 mm chromium-cobalt alloy polyaxial screws, used with the ChM 5.0 ChLP titanium plates, exhibit diminished screw stability as off-axis insertions increase. Despite a relatively high ATE after multiple insertions of the screws, the load-to-failure of the screw head and the plate hole interface was further compromised. Depending on the particular system`s specification, surgeons should minimize the off-axis placement of polyaxial screws and avoid reintroducing these screws in the same hole, as a reduction in the screw bending strength could be expected.

## Supporting information

S1 Dataset (XLSX)
